# Genome-wide association study provides insights into genes related with horn development in Nelore beef cattle

**DOI:** 10.1371/journal.pone.0202978

**Published:** 2018-08-30

**Authors:** Nedenia Bonvino Stafuzza, Rafael Medeiros de Oliveira Silva, Elisa Peripolli, Luiz Antônio Framartino Bezerra, Raysildo Barbosa Lôbo, Cláudio de Ulhoa Magnabosco, Fernando A. Di Croce, Jason B. Osterstock, Danísio Prado Munari, Daniela A. Lino Lourenco, Fernando Baldi

**Affiliations:** 1 Departamento de Ciencias Exatas, Faculdade de Ciencias Agrarias e Veterinarias (FCAV), Universidade Estadual Paulista Julio de Mesquita Filho (UNESP), Jaboticabal, Sao Paulo, Brazil; 2 Department of Animal and Dairy Science, University of Georgia, Athens, Georgia, United States of America; 3 National Center for Cool and Cold Water Aquaculture (NCCCWA), Agricultural Research Service (ARS), United States Department of Agriculture (USDA), Leetown, West Virginia, United States of America; 4 Departamento de Zootecnia, Faculdade de Ciencias Agrarias e Veterinarias (FCAV), Universidade Estadual Paulista Julio de Mesquita Filho (UNESP), Jaboticabal, Sao Paulo, Brazil; 5 Departamento de Genetica, Faculdade de Medicina de Ribeirao Preto (FMRP), Universidade de Sao Paulo (USP), Ribeirao Preto, Sao Paulo, Brazil; 6 Associaçao Nacional dos Criadores e Pesquisadores (ANCP), Ribeirao Preto, Sao Paulo, Brazil; 7 Empresa Brasileira de Pesquisa Agropecuaria (EMBRAPA), Planaltina, Distrito Federal, Brazil; 8 Zoetis, Kalamazoo, Michigan, United States of America; National Cheng Kung University, TAIWAN

## Abstract

The causal mutation for polledness in Nelore (*Bos taurus indicus*) breed seems to have appeared first in Brazil in 1957. The expression of the polled trait is known to be ruled by a few groups of alleles in taurine breeds; however, the genetic basis of this trait in indicine cattle is still unclear. The aim of this study was to identify genomic regions associated with the hornless trait in a commercial Nelore population. A total of 107,294 animals had phenotypes recorded and 2,238 were genotyped/imputed for 777k SNP. The weighted single-step approach for genome-wide association study (WssGWAS) was used to estimate the SNP effects and variances accounted for by 1 Mb sliding SNP windows. A centromeric region of chromosome 1 with 3.11 Mb size (BTA1: 878,631–3,987,104 bp) was found to be associated with hornless in the studied population. A total of 28 protein-coding genes are mapped in this region, including the taurine *Polled* locus and the *IFNAR1*, *IFNAR2*, *IFNGR2*, *KRTAP11-1*, *MIS18A*, *OLIG1*, *OLIG2*, and *SOD1* genes, which expression can be related to the horn formation as described in literature. The functional enrichment analysis by DAVID tool revealed cytokine-cytokine receptor interaction, JAK-STAT signaling, natural killer cell mediated cytotoxicity, and osteoclast differentiation pathways as significant (P < 0.05). In addition, a runs of homozygosity (ROH) analysis identified a ROH island in polled animals with 2.47 Mb inside the region identified by WssGWAS. Polledness in Nelore cattle is associated with one region in the genome with 3.1 Mb size in chromosome 1. Several genes are harbored in this region, and they may act together in the determination of the polled/horned phenotype. Fine mapping the locus responsible for polled trait in Nelore breed and the identification of the molecular mechanisms regulating the horn growth deserve further investigation.

## Introduction

Brazil has the world’s largest commercial beef cattle herd, which is estimated at 215.2 million head [1]. The vast majority of Brazilian beef cattle production is based in extensive grazing systems in challenging environments due to tropical climate. Because of their resistance to parasites, adaptability towards the tropical climate and extensive production systems, indicine breeds are widely used in Brazilian beef cattle production systems [2].

The Nelore (*Bos taurus indicus*) breed, derived from Ongole, Hariana and other Indian breeds [3], is the most important beef cattle breed in Brazil, representing around 80% of the total head. The genetic makeup of Nelore breed in Brazil is mainly the result of less than 7,000 animals imported from India from the end of the 19th century up until 1963, when the importation of animals, embryos and semen was banned [3]. The herd multiplied quickly, mainly due to its high rates of productive and reproductive performance in tropical climates [4].

The intensive use of few animals for reproduction and the biotechnologies employed to speed up genetic progress have been contributing to the decreased genetic variability of the Nelore breed [5]. In studying the genetic makeup of breeding programs for Nelore in Brazil, Magnabosco et al. [6] identified that the majority were descendants of six main sires named Karvadi, Taj Mahal, Kurupathy, Golias, Godhavari, and Rasta.

According to Santiago [3], in 1957 a purebred Brazilian Nelore male, named Caburey, was born without horns (referred to as “polled”). Since there is no description in the literature about naturally polled Nelore in other countries, the natural absence of horns in Nelore cattle seems to have emerged in Brazil. Caburey was intensively used in matings with many Nelore purebred cows and all of his offspring were polled, propagating this trait in a large herd. The Nelore herd book was implemented in Brazil in 1938, and in 1969 the Brazilian Association of Zebu Cattle Breeders (ABCZ) registered Caburey as the first polled Nelore.

The cattle horn consists of a pneumatized bone core fused with the skull frontal bone and is covered by a continually growing keratin sheath. This structure results from the differentiation and remodeling of various tissues and its interaction [7, 8]. There is a worldwide trend for eliminating horns to avoid accidents related to herd management. In this context, the presence of naturally polled Nelore is beneficial for the beef industry and the underlying genetics can be exploited to avoid dehorning, an expensive procedure also associated with pain, stress, and weight loss for the animals. Because the presence/absence of horns has a strong genetic background, matings can be arranged to increase the incidence of the polled alleles. In fact, the number of genetically polled Nelore in commercial herds is rapidly increasing in Brazil. In the last 10 years, approximately 5.3% of the Nelore cattle registered by the Brazilian Association of Zebu Cattle Breeders were polled [9].

Inheritance of horns was one of the first Mendelian traits reported in cattle, although it later became evident that the inheritance pattern is more complex than a single gene mutation initially reported by Bateson and Saunders [10]. The genetic cause of hornless cattle has been more thoroughly investigated in taurine (*Bos taurus taurus*) [7, 11, 12] than in indicine cattle breeds (*Bos taurus indicus*). The most commonly accepted model for inheritance of horns describes three loci, each with two alleles, controlling the phenotype development: 1. The *Polled* locus with *P* (polled) allele dominant to *p* (horned) allele; 2. The *Scurs* locus with *Sc* (development of scurs) and *sc* (absence of scurs) alleles; 3. The *African horn* locus with *Ha* (horned) and *ha* (polled) alleles. Nevertheless, no experiment has been able to fully elucidate the mechanism of inheritance of horns in cattle, and most of the studies refer to this trait in European cattle (*Bos taurus taurus*) [13–15].

Although the *Polled* mutation has been mapped to the centromeric region of the bovine chromosome 1 (BTA1) in many taurine breeds [11, 12, 16–18], the lack of a fine characterization of this locus has not allowed the identification of a candidate gene responsible for this trait. Two different candidate neomutations on BTA1 have been recently accepted as the genetic cause of hornless in taurine cattle: a complex allele of Friesian origin described as an 80,128 bp duplication (1,909,352–1,989,480 bp), and a simple allele of Celtic origin described as a duplication of 212 bp (1,705,834–1,706,045 bp) in place of a 10-bp deletion (1,706,051–1,706,060 bp) [11, 16, 17]. Carlson et al. [19], using genome editing technologies, performed the first empirical validation of a putative causative allele for Polled in the Holstein breed by the introgression of the putative *Celtic Polled* allele into the genome of a cattle embryo fibroblasts to generate hornless animals.

Genetically hornless cattle occasionally grow scurs between 4–18 months of age [20]. Scurs are small corneous structures that develop in the same area as horns but are not firmly attached to the skull. The most recent inheritance model assumes the existence of a *Scurs* locus on BTA19, but the genetic heterogeneity is evident in some [20–23]. The inheritance pattern of the *Scurs* locus is described as sex-influenced, where the allele Sc is dominant in males and recessive in females, although exception to this model have been described as reviewed by [22].

It is hypothesized that indicine animals present an additional locus (*African horn*) controlling horn development [24, 25]. This postulated locus, which is believed to be segregating independently, but with epistatic effect on *Polled* locus [14], has not been mapped to a bovine chromosome [26]. The sex-influenced effect of the *Scurs* loci and the epistatic effect of the *African horn* loci apparently do not modify the horn shape in horned animals (*p/p*) [27].

Furthermore, although the studies of inheritance of horns aim to detect presence or absence of horns, other traits such as size, shape, and orientation of horns could be under the influence of many genes, each with minor effects like observed in any other quantitative trait [25, 28].

Because the causal mutation responsible for the polled trait in Nelore was first reported in a Brazilian herd, the aim of this study was to perform a genome-wide association study (GWAS) to identify the genomic region, potential candidate genes and their biological mechanisms underlying polledness in Nelore beef cattle.

## Material and methods

### Ethics statement

The use of animals, including welfare, husbandry, experimental procedures, and the collection of blood samples used for this study, was approved by the ethics committees belonging to the Brazilian National Association of Breeders and Researchers (ANCP), Ribeirao Preto, SP, Brazil and the School of Agricultural and Veterinarian Sciences (FCAV)—Sao Paulo State University (UNESP), Jaboticabal, SP, Brazil.

### Animals and genotyping

The data set used in this study was provided by the National Association of Breeders and Researchers (ANCP), Ribeirao Preto, SP, Brazil. The data contained information from 18 Nelore herds, from the southeast and mid-west regions of Brazil, which participate in the ANCP breeding program. Pedigree information was available on 202,717 animals, of which 107,294 had phenotypes recorded for the polled trait (92,625 horned and 14,669 polled). Horned animals were recorded as 1, whereas polled animals were in category 2.

A total of 963 animals were genotyped with the BovineHD BeadChip (777k; Illumina, San Diego, CA). They were used as reference for genome imputation of 1,365 animals that were genotyped with CLARIFIDE^®^ Nelore 2.0 (12k; Zoetis, Kalamazoo, MI). Imputation was performed using the FImpute software [29]. Only SNPs on autosomal chromosomes with defined position according to the UMD_3.1 bovine genome assembly were used in the analyses. The genotype quality control (QC) excluded SNPs with unknown genomic position according to the UMD_3.1 bovine genome assembly, located on sex chromosomes, monomorphic, with minor allele frequency (MAF) less than 5% and call rate lower than 90%. Samples with a call rate lower than 90% were also excluded. After quality control, genotypes on 461,865 SNPs were available for 2,238 animals, of which 1,721 had phenotypes recorded.

### Weighted single step genome wide association study (WssGWAS)

The WssGWAS methodology allow us to use all the information available (genotyped and ungenotyped animals) in one-step procedure. Because the polled trait is recorded as categorical, a threshold model was used for GWAS. Genomic breeding values were estimated using the single-step genomic BLUP (ssGBLUP) approach, which combines all relationship information available (based on genotypes and pedigree) into a single matrix (**H**^**-1**^). The model assumed an underlying distribution for the analyzed trait and can be represented as follows:

I=1μ+Xa+e (1)

where **I** is the vector of underlying distribution of polledness; **μ** is the general mean, **a** is the vector of additive direct genetic effects, **1** is a vector of ones, and **X** is the incidence matrix that relates animals with phenotypes. The animal effects of the genotyped (***a***_***g***_) animals were estimated as described by Wang et al. [30] ***a***_***g***_ = ***Zu***, where ***Z*** was a matrix that related genotypes of each locus and ***u*** is a vector of SNP effects. The variance of animal effects was assumed as:

$$var(a_{g})=var\;(Zu)=ZDZ^{\prime }\sigma_{u}^{2}=G^{*}\sigma_{a}^{2}$$ (2)

where ***D*** is a diagonal matrix of weights for variances of SNP (***D* = *I***), $\sigma_{u}^{2}$ is the genetic additive variance captured by each SNP when no weights are present, and G* is the weighted genomic relationship matrix.

The ratio of covariance of additive genetic (***a***_***g***_) and SNPs (***u***) effects is:

$$var\;\lfloor \begin{array}{c} a_{g}\\ u \end{array}\rfloor =[\begin{array}{cc} ZDZ\prime & ZD\prime \\ DZ\prime & D \end{array}]\;\sigma_{u}^{2}$$ (3)

sequentially:

$$G^{*}=\frac{var\;(a_{g})}{\sigma_{a}^{2}}=\frac{var\;(Zu)}{\sigma_{a}^{2}}=ZDZ\prime \lambda$$ (4)

where ***λ*** is a normalizing constant described by VanRaden [31] as:

$$\lambda =\frac{\sigma_{u}^{2}}{\sigma_{a}^{2}}=\frac{1}{\sum_{i=1}^{m}{2p}_{i}\;(1-p_{i})}$$ (5)

where ***m*** is the number of SNPs and ***p***_***i***_ is the frequency of the second allele in the ***i***-th SNP. The SNP effects can be described by Wang et al. [30]:

$$\hat{u}=\lambda DZ\prime G^{*-1}{\hat{a}}_{g}=DZ\prime {\left[ZDZ^{\prime }\right]}^{-1}\;{\hat{a}}_{g}$$ (6)

The estimated SNP effects can be used to calculate the variance of each individual SNP [32], which can be used as different weighting for each SNP:

$${\hat{\sigma }}_{\hat{u},i}^{2}={\hat{u}}_{i}^{2}\;2p_{i}\;(1-p_{i})$$ (7)

The following iterative process was used to calculate SNP effect and variance [30]: 1. Set **D** = **I**; 2. Construct the genomic relationship matrix (**G)** as described by VanRaden [31]; 3. Calculate genomic estimated breeding values (GEBV) for all animals in the data set using ssGBLUP; 4. Calculate the SNP effect; 5. Calculate the variance of each SNP; 6. Normalize **D** to keep the additive genetic variance constant; 7. Iterate from step 2. The results were obtained after one loop to step 2. The analyses were performed using THRGIBBS1F90 software [33], and results were presented for 1 Mb sliding SNP-windows.

The percentage of the additive genetic variance explained by ***i***^th^ region was calculated as followed [34]:

$$\frac{var(a_{i})}{\sigma_{a}^{2}}=x\;100=\frac{var\;(\sum_{j=i}^{1Mb}z_{j}{û}_{j})}{\sigma_{a}^{2}}\;x\;100$$ (8)

where ***a***_***i***_ is genetic value of the ***i***-th region that consists of 1Mb window length physical size, $\sigma_{a}^{2}$ is the total genetic variance, ***Z***_***j***_ is vector of SNP content of the ***j***-th SNP for all individuals, and ${û}_{j}$ is effect of the ***j***-th SNP within the ***i***-th region. The windows explaining more than 1% of the additive genetic variance were explored for possible loci related with horn development.

The analysis consisted of a single chain of 300,000 cycles considering fixed variance components (h^2^ = 0.99 ± 0.000028) previously estimated. The estimation of SNP effects and variances was done by the postGSf90 software [35].

### Gene prospection and functional enrichment analysis

The chromosome segments that explained more than 1% of the additive genetic variance were further explored for possible loci related with horn development. The Ensembl BioMart tool (http://www.ensembl.org/biomart/) was used for the identification of genes using the *Bos taurus* UMD_3.1 genome assembly.

The classification of genes regarding their biological function was performed by the Database for Annotation, Visualization and Integrated Discovery (DAVID) v. 6.8 [36, 37] using all annotated genes in the bovine genome as background. Gene Ontology (GO) biological process, GO cellular component and GO molecular function annotation data sets were used for functional enrichment analysis considering a P value < 0.05 threshold for significance.

### Runs of homozygosity (ROH)

PLINK v1.90 software [38], which uses a sliding window approach to scan along each genotype at each SNP marker position to detect homozygous segments [39], was used to identify runs of homozygosity (ROH) islands on BTA1. A ROH was defined according to the following parameters (i) a sliding window of 50 SNPs; (ii) a proportion of 0.05 homozygous overlapping windows; (iii) a minimum of 100 consecutive SNPs included in a ROH; (iv) a minimum length of 1 Mb for a ROH; (v) a maximum gap of 0.5 Mb between consecutive homozygous SNPs; (vi) a density of one SNP per 50 Kb; and (vii) up to one heterozygous genotype were allowed in a ROH. ROH were defined by a minimum of 1 Mb in length to avoid short and common ROH that may occur due to linkage disequilibrium (LD) [40]. ROH islands on BTA 1 were defined as regions where SNPs were outliers according to boxplot distribution and regions displaying at least 100 consecutive outliers SNPs were then classified as an island.

## Results and discussion

**Fig 1:**
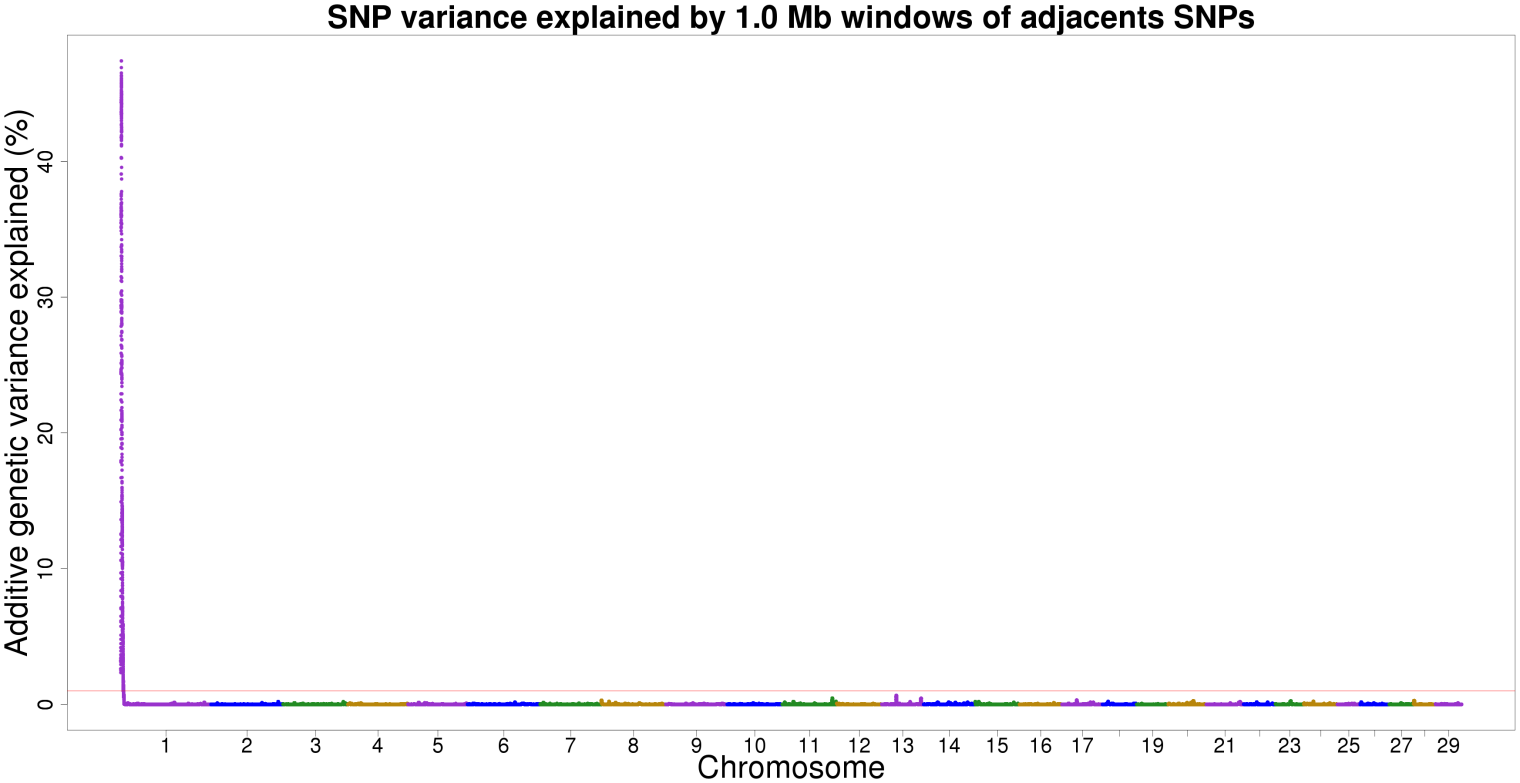
Proportion of additive genetic variance, explained by 1 Mb windows of adjacent SNPs, for the polled trait in Nelore cattle.

A total of three adjacent windows were found to be associated with the polled trait in Nelore beef cattle, represented by one peak on BTA1 formed by 824 SNPs (Fig 1). Despite being distributed in three windows, these windows were very close and all the SNPs were located in a 3.11 Mb centromeric region (between 878,631 and 3,987,104 bp), representing 1.95% of the chromosome total length (158.34 Mb).

The SNP windows that accounted for more than 1% of the genetic variance were used to search for candidate genes, represented in Table 1. A total of 37 genes (28 protein-coding genes, eight non-coding RNAs, and one transfer RNA) and the taurine *Polled* locus were identified in three windows on BTA1 that explained 65.54% of this trait in Nelore breed. The taurine *Polled* locus present two alleles described as responsible for the polled phenotype in taurine breeds: a duplication of 212 bp (BTA1: 1,705,834–1,706,045 bp) in place of a 10-bp deletion (BTA1: 1,706,051–1,706,060 bp) called Celtic allele and an 80,128 bp duplication (BTA1: 1,909,352–1,989,480 bp) named Friesian allele [11, 16, 17].

**Table 1:** Identification of genes based on BTA1 based on the additive genetic variance (Var) explained by 1 Mb windows of adjacent SNPs of the polled trait.

Position (bp)	SNPs	Genes	Var
878,631–1,873,922	247	*ATP5O*^1^, *ITSN1*^1^, *TRNAC-GCA*^2^, *CRYZL1*^1^, *DONSON*^1^, *SON*^1^, *GART*^1^, *LOC104970777*^3^, *DNAJC28*^1^, *TMEM50B*^1^, *IFNGR2*^1^, *IFNAR1*^1^, *LOC104970778*^3^, *IL10RB*^1^, *IFNAR2*^1^, *LOC526226*^1^, *OLIG1*^1^, *LOC100848368*^3^, *OLIG2*^1^	47.18%
1,983,902–2,983,314	329	*C1H21orf62*^1^, *PAXBP1*^1^, *SYNJ1*^1^, *C1H21orf59*^1^, *EVA1C*^1^, *LOC104970779*^3^, *LOC104970780*^3^, *URB1*^1^, *MRAP*^1^, *MIS18A*^1^, *HUNK*^1^	14.66%
2,987,912–3,987,104	248	*SCAF4*^1^, *SOD1*^1^, *TIAM1*^1^, *MIR2284I*^3^, *MIR2284X*^3^, *LOC101906247*^3^, *KRTAP11-1*^1^	3.70%

^1^protein-coding gene,

^2^transfer RNA,

^3^non-coding RNA.

Within the first window identified on BTA1 (8978,631–1,873,922 bp) that explained 47.18% of the genetic variance, a total of 15 protein-coding genes were identified, some having been associated with the polled trait in taurine breeds. The *IFNAR1* (interferon alpha and beta receptor subunit 1) and *IFNAR2* (interferon alpha and beta receptor subunit 2) genes encode type I membrane proteins that form the two chains of a receptor for interferons alpha (*IFN-α*) and beta (*IFN-β*). Interferon-alfa/beta (*IFN-α/β*) signaling is physiologically critical for the maintenance of bone mass through negative regulation of osteoclastic bone resorption [41]. Medugorac et al. [16], in a study with 31 taurine breeds, described a 202-bp-indel located between *IFNAR2* and *OLIG1*, and one haplotype that included *IL10RB*, *IFNAR2*, *OLIG1*, *C1H21orf62* and *GCFC1*, as responsible for polledness in several taurine breeds.

The *OLIG1* (oligodendrocyte transcription factor 1) and *OLIG2* (oligodendrocyte transcription factor 2) genes encode basic helix–loop–helix (bHLH) transcription factors known to regulate oligodendrocyte differentiation and maturation in the developing nervous system [42]. Allais-Bonnet et al. [43] identified that the *OLIG2* genes play a role in horn ontogenesis of taurine breeds by qPCR analysis. Seichter et al. [17] identified in nine taurine breeds a 381-kb interval on BTA1 associated with polled phenotype, which contain *HIST1H4C*, *OLIG1* and *C1H21orf62* genes.

The *IFNGR2* (interferon gamma receptor 2) gene encodes the non-ligand-binding beta chain of the gamma interferon receptor which is involved in immunological pathways. Glatzer et al. [44] identified the SNP AC000158: g.1390292G>A within intron 3 of *IFNGR2* gene in perfect co-segregation with the polled trait in Holsteins. However, results from another Holstein population disagree with the perfect association between a SNP within intron 3 of the *IFNGR2* gene and polledness [11]. There are currently no known functions of the *IFNGR2* gene that could play a role in the growth of horns.

The second window (BTA1: 1,983,902–2,983,314 bp), which explained 14.66% of genetic variance, contained nine protein-coding genes. The *SYNJ1* (synaptojanin 1) gene encodes a protein that regulates levels of membrane phosphatidylinositol-4,5-bisphosphate, acting on synaptic transmission and membrane trafficking in nervous system [45]. Cargill et al. [18] identified SNPs in *SYNJ1*, *IFNAR2*, and *C21orf59* genes as concordant with the polledness trait in Holstein breed. However, Wöhlke et al. [46] discarded the hypothesis that SNPs in *SYNJ1* gene could be responsible for polledness in German Holstein, Limousin, Charolais and Pinzgauer cattle breeds.

The *MIS18A* (MIS18 kinetochore protein A) gene codes for a critical protein in the proliferation of keratinocytes and stratification of the epidermis [47]. The barrier function of the skin is maintained, at least in part, by the continuous proliferation and stratification of epidermal keratinocytes [48, 49].

A total of four protein-coding genes were identified on the third window that explained 3.70% of genetic variance (2,987,912–3,987,104 bp). The *SOD1* (superoxide dismutase 1) gene encodes a soluble cytoplasmic protein that act as a homodimer to convert naturally-occurring but harmful superoxide radicals to molecular oxygen and hydrogen peroxide, destroying free superoxide radicals in the body. Nojiri et al. [50] demonstrated that *SOD1* knockout mice (*SOD1*^-/-^) exhibit the induction of intracellular reactive oxygen species (ROS) and bone fragility resulting from low-turnover bone loss and impaired collagen cross-linking. This study provided evidences that *SOD1* regulates the viability and function of osteoblasts during osteoclastogenesis through cytoplasmic superoxide metabolism and RANKL/M-CSF signaling.

The same window also includes *MIR2284I* and *MIR2284X*, which encode bta-mir-2284i and bta-mir-2284x miRNA, respectively. According to miRbase (http://www.mirbase.org/), both miRNA sequences overlap with *TIAM1* (T-cell lymphoma invasion and metastasis 1) gene transcripts. The *TIAM1* gene encodes a RAC1-specific guanine nucleotide exchange factor (GEF) which regulates RAC1 mediated signaling pathways that affect cell shape, polarity, growth, migration, adhesion, and survival, and as well as influencing actin cytoskeletal formation, endocytosis, and membrane trafficking. This gene also plays an important role in both apoptotic and pro-apoptotic mechanisms [51, 52]. The *TIAM1* gene is located in the same window as *MIR2284I* and *MIR2284X* (3,244,597–4,226,867 bp).

A cluster of keratin-associated proteins (*KRTAP*) were identified at the end of third window, described as some key structural components of the wool/hair fiber that form the matrix and that cross-link the keratin intermediate filaments [53] determining the physical properties of hair fiber. The *KRTAP11-1* (keratin associated protein 11) gene codes for a hair keratin-associated protein which may play an important role on keratin-bundle assembly in the wool/hair cortex [54, 55].

A phenotype-genotype association study was performed with 481 Nelore bulls using single-marker regression under a mixed model framework and identified 295 SNPs significantly associated (P < 1.07×10^−7^) with absence of horns [56]. These SNPs were represented by two peaks that cover genomic regions in the beginning (~78.66 Kb) and in the middle (~60.66 Mb) of BTA1 that include the *Polled* locus described in taurine breeds and some candidate genes already described for these trait in taurine breeds, such as *OLIG2*, *FOXL2* and *RXFP2*. The same authors identify the second significant peak as a misassembled segment on BTA1 (60,578,448–60,664,293 bp) and they re-located it closer to the *Polled* locus (4,008,972–5,968,730 bp), showing that assembly errors could be an important contributor to the ongoing dispute regarding the candidate gene and causal variant underlying the polled trait. Looking carefully at the results presented, we identified a smaller significant region with only 3.11 Mb (0,87–3,98 Mb) using a different method (WssGWAS).

Although our results not allow corroborate with the hypothesis that indicine breeds present an additional locus (*African horn*) controlling horn development [24, 25] because our study was performed using SNP data, we refined the region where the locus controlling horn development in Nelore cattle is located.

### Runs of homozygosity

The BTA1 was scanned for ROH as a complementary strategy to WssGWAS. A total of five ROH islands were revealed: 269,100–2,739,000 bp, 30,950,000–32,130,000 bp, 39,420,000–42,160,000 bp, 43,680,000–44,080,000 bp, and 58,900,000–60,580,000 bp. The first ROH island identified (269,100–2,739,000 bp) overlapped with that 3.11 Mb region identified by the WssGWAS (Table 1), providing a consensus and more refined region for hornless trait in Nelore breed with 1.86 Mb size (878,631–2,739,000 bp).

Although several studies on taurine breeds selected for milk and meat production have shown the greatest number of ROH on BTA1 [40, 57, 58], none of them described their physical position within the chromosome, precluding comparisons.

### Functional enrichment analysis

The gene-annotation enrichment analysis by DAVID tool identified the type I interferon receptor activity (GO:0004905), and cytokine receptor activity (GO:0004896) as the most significant (P < 0.05) GO terms for molecular functions. The type I interferon signaling pathway (GO:0060337) was the most significant GO term related to biological processes while plasma membrane (GO:0005886) was the most significant GO term for cellular component.

A total of four significant (P < 0.05) KEGG pathways were identified: cytokine-cytokine receptor interaction (bta04060), JAK-STAT signaling pathway (bta04630), natural killer cell mediated cytotoxicity (bta04650), and osteoclast differentiation (bta04380). The *IFNAR1*, *IFNAR2*, *IFNGR2*, and *IL10RB* genes act on cytokine-cytokine receptor interaction (bta04060) and JAK-STAT signaling pathway (bta04630), while *IFNAR1*, *IFNAR2*, *IFNGR2* genes also participate in natural killer cell mediated cytotoxicity (bta04650) and osteoclast differentiation (bta04380) pathways. These four genes were identified in the same window (BTA1: 878,631–1,873,922 bp) that explains 47.18% of the trait.

The four pathways identified in this study have connections and interact with each other. The skeletal and immune systems have many regulatory molecules in common including cytokines and transcription factors. Furthermore, cells from immune system are produced in the bone marrow, interacting with bone cells [59].

The development and homeostasis of the vertebrate skeletal system depend on a critical balance between osteoblast (bone formation) and osteoclasts (bone resorption) [60, 61]. This balance must be tightly controlled by various regulatory systems including the endocrine, nervous, and immune systems [59]. Osteoclasts are multinucleated cells originating from the fusion of mononuclear progenitors of hematopoietic monocyte-macrophage lineage which are responsible for bone resorption, playing a central role in the formation of the skeleton and regulation of bone mass [62]. The major function of osteoclasts is to hydrolyze inorganic hydroxyapatite and degrade organic bone matrix, mainly collagen [63].

Although several signaling pathways have been identified as controlling bone homeostasis through the interplay between osteoclasts and osteoblasts [41, 54, 64–67] the *RANK/RANKL/OPG* (osteoprotegerin) is the best-established regulatory mechanism controlling osteoclast development and function, which should be strictly regulated to maintain bone homeostasis [64, 68]. Takayanagi et al. [41] showed that *RANKL* gene induces the *IFNB1* gene in osteoclast precursor cells inhibiting the differentiation by interfering with the *RANKL*-induced expression of c-FOS, a pivotal transcription factor for the development of osteoclasts. These authors showed the importance of this regulatory mechanism for bone homeostasis after observing that *IFNAR1* knockout mice (*IFNAR1*^-/-^) display severe reduction of bone mass (osteopenia) accompanied by the activation of osteoclastogenesis.

Cytokines are soluble extracellular proteins or glycoproteins that regulate numerous aspects of inflammatory host defenses, angiogenesis, cell growth, differentiation, and cell death. Cytokines are released by various cells in the body, usually in response to an activating stimulus, and they induce responses through binding to specific receptors on the cell surface of target cells. Many cytokines mediate aspects of hematopoiesis and immune response through activation of the JAK-STAT signaling pathway [69] and regulate the osteoclast formation and function through modulating the *RANKL* gene expression by osteoblasts/stromal cells [60].

The JAK-STAT signaling pathway (bta04630) plays an important role in the growth and differentiation of a variety of cell types. Although the function of the JAK-STAT pathway in musculoskeletal system has not been well characterized, increasing evidence suggests that this pathway may be involved in bone development, metabolism and homeostasis as reviewed by [70].

## Conclusion

For the first time we described networks of genes that could be involved in horn development in Nelore beef cattle. Polledness in Nelore cattle is associated with one 3.11 Mb region in chromosome 1. Several genes are harbored in this region, and they may act together in the determination of the polled/horned phenotype. In addition, we suggest that the postulated *African horn* locus is located very close to the *Polled* locus described for taurine breeds or it may be a mutation in the same region, since the literature that suggests the existence of an *African horn* locus is based on inconsistent inheritance patterns and dates from the last century.

Considering that the causal mutation responsible for polledness in Nelore appears to have emerged in Brazil, this study offers a path to fine map the *Polled* locus and to identify the molecular mechanisms regulating the growth of horns in Nelore beef cattle. Furthermore, this study offers a promising approach to further investigate the locus responsible for the polled trait in the Nelore breed through re-sequencing. Based on that, it will be possible to identify some misassembled segments and to obtain a better annotation of this genomic region that has economic importance in cattle.
